# Mitotic Arrest and Apoptosis in Breast Cancer Cells Induced by *Origanum majorana* Extract: Upregulation of TNF-α and Downregulation of Survivin and Mutant p53

**DOI:** 10.1371/journal.pone.0056649

**Published:** 2013-02-22

**Authors:** Yusra Al Dhaheri, Ali Eid, Synan AbuQamar, Samir Attoub, Mohammad Khasawneh, Ghenima Aiche, Soleiman Hisaindee, Rabah Iratni

**Affiliations:** 1 Department of Biology, College of Science, United Arab Emirates University, Al-Ain, United Arab Emirates; 2 Department of Pharmacology & Therapeutics, Faculty of Medicine & Health Sciences, United Arab Emirates University, Al-Ain, United Arab Emirates; 3 Department of Chemistry, College of Science, UAE University, United Arab Emirates University, Al-Ain, United Arab Emirates; 4 Département de Biochimie et Microbiologie, Faculté des sciences biologiques et agronomiques, Université Mouloud Mammeri, Tizi-Ouzou, Algeria; University of Medicine and Dentistry of New Jersey, United States of America

## Abstract

**Background:**

In the present study, we investigated the effect of *Origanum majora*na ethanolic extract on the survival of the highly proliferative and invasive triple-negative p53 mutant breast cancer cell line MDA-MB-231.

**Results:**

We found that *O. majorana extract* (OME) was able to inhibit the viability of the MDA-MB-231 cells in a time- and concentration-dependent manner. The effect of OME on cellular viability was further confirmed by the inhibition of colony growth. We showed, depending on the concentration used, that OME elicited different effects on the MDA-MB 231 cells. Concentrations of 150 and 300 µg/mL induced an accumulation of apoptotic–resistant population of cells arrested in mitotis and overexpressing the cyclin-dependent kinase inhibitor, p21 and the inhibitor of apoptosis, survivin. On the other hand, higher concentrations of OME (450 and 600 µg/mL) triggered a massive apoptosis through the extrinsic pathway, including the activation of tumor necrosis factor-α (TNF-α), caspase 8, caspase 3, and cleavage of PARP, downregulation of survivin as well as depletion of the mutant p53 in MDA-MB-231 cells. Furthermore, OME induced an upregulation of γ-H2AX, a marker of double strand DNA breaks and an overall histone H3 and H4 hyperacetylation.

**Conclusion:**

Our findings provide strong evidence that *O. majorana* may be a promising chemopreventive and therapeutic candidate against cancer especially for highly invasive triple negative p53 mutant breast cancer; thus validating its complementary and alternative medicinal use.

## Introduction

Breast cancer is the most frequently diagnosed cancer among women and ranks second as a cause of cancer death in women after lung cancer. An estimated 226,870 new cases of invasive breast cancer are expected to occur among women in the US during 2012 [1]. Plants have been shown to be an excellent source of new drugs, including anticancer agents. Identification and development of new chemotherapeutic agents from plants have gained significant recognition in the field of cancer therapy and become a major area of experimental cancer research. The majority of the chemotherapeutic drugs used in cancer treatment, is either from plant origin or chemically-altered plant products and phytochemicals [2]. In fact, plant-derived anticancer drugs are much more effective and do not have large side-effect consequences compared to synthetic drugs. Examples of anticancer drugs derived from plants and currently in clinical use include the *vinca* alkaloids vinblastine and vincristine were isolated from *Catharan roseus*, the terpene paclitaxel from *Taxus brevifolia* Nutt., and the DNA topoisomerase I inhibitor camptothecin from *Camptotheca acuminata* [3].

Phytochemicals exert their chemoprevention effect of carcinogenesis through several mechanisms. These include inhibition of genotoxic effects, increased antioxidants and anti-inflammatory activity, modulation of cellular signaling pathways and altering gene expression to inhibit cell proliferation and/or induce apoptosis [4].

It is well known that cancer is pathological condition that has been associated with aberrantly regulated apoptosis. It is currently accepted that certain phytochemicals and whole plant extracts can affect the overall process of carcinogenesis by multiple mechanisms. Since apoptosis provides a physiologic protective mechanism for eliminating genetically damaged cells, initiated cells or cells progressed to malignancy; phytochemicals affecting apoptosis can have an important effect on carcinogenesis [5].

Increasing number of studies have shown evidence of chemoprevention and chemotherapy by stimulating apoptosis in pre-cancerous and cancerous cells *in vitro* or *in vivo* suggesting that apoptosis is likely to be a crucial mechanism to suppress carcinogenesis. Epigallocatechin gallete (EGCG) (from green tea), curcumin (*Curcuma longa*), quercetin (Vegetable and fruits) are some examples of chemopreventive agents, from natural origin, that induce apoptosis in carcinogenesis models or in human chemoprevention trials [6], [7]. Also, a number of plant extracts, such as blueberry [8], mushroom [9], *gingerol* [10], to name just a few, were shown to have anticancer effects against breast cancer cells.

*Origanum majorana* belongs to the family Lamiaceae. It is commonly known as marjoram. It is a perennial herb and widespread worldwide. A large number of known species of the genus *Origanum* are utilized worldwide as spices and flavoring agents and has a long history of both culinary and medicinal use. *O*. *majorana* is used as a home remedy for chest infection, cough, sore throat, rheumatic pain, nervous disorders, stomach disorders, cardiovascular diseases, and skin care [11], [12]. Many of such traditional uses of *marjoram* species were confirmed in several studies utilizing both *in vitro* and *in vivo* approaches.

Several reports indicate that *O. majorana* is very rich in phenolic compounds. The high phenolics content in *Origanum* has a capacity to scavenge free radicals and shown to be associated with the strong antioxidant activity [13]. *O. marjorana* was shown to contain phenolic terpenoids (thymol and carvacrol), flavonoids (diosmetin, luteolin, and apigenin), tannins, hydroquinone, phenolic glycosides (arbutin, methyl arbutin, vitexin, orientin, and thymonin) and triterpenoids (ursolic acid and oleanolic acid) [14].

*O. majorana* has been reported to exhibit a significant anti-microbial activity [15]. Several studies have also demonstrated that ethanolic, aqueous extracts and essential oil of *O. majorana* could protect against liver and kidney damage and genotoxicity induced by lead acetate [16]–[18]. *O. majorana* has also been found to inhibit platelet adhesion aggregation and secretion [19]. Furthermore, it has been shown that this plant exerts a low cytotoxicity on several hepatoma cell lines [20]. It has been shown by Al Harbi that extract of *O. majorana* reduced the side effects induced by cyclophosphamide, an established anticancer drug, without altering its cytotoxicity [12].

In the present study, we investigated the effect of *Origanum majorana* ethanolic extract (OME) on breast cancer cells. We examined the effects of OME on cell viability, cell cycle, apoptosis, and the levels of several cell cycle and apoptosis control proteins in the highly proliferative and invasive Estrogen Receptor (ER)-negative, mutant p53 breast cancer cell lines MDA-MB-231. Our results demonstrate that OME can inhibit the growth of the MDA-MB-231 cells by causing cell cycle arrest and apoptosis dependent on the downregulation of survivin and mutant p53.

## Materials and Methods

### Preparation of the *Origanum majorana* Ethanolic Extract

*Origanum marjorana* commonly known as “marjoram” and used as a culinary herb, was obtained from a private commercial farm located in the Tyre region of Lebanon. All necessary permits were obtained for the harvesting of the leaves. The identity of the OM dried leaves used in this study was further confirmed by a plant taxonomist. 5.0 g of the dried leaves were ground to a fine powder using a porcelain mortar and pestle. The powder was suspended in 100 mL of 70% absolute ethanol and the mixture was kept in the dark for 72 hours at 4°C in a refrigerator without stirring. The mixture was then filtered through a glass sintered funnel and the filtrate was evaporated to dryness using a rota-vapor at room temperature. The green residue was kept under vacuum for 2–3 hours and its mass was recorded.

### Cell Culture and Reagents

Human breast cancer cells MDA-MB-231 were maintained in DMEM (Hyclone). The culture media was supplemented with 10% fetal bovine serum (invitrogen), 100 U/ml penicillin/streptomycin (invitrogen). Antibodies to p21 (556431), PARP (556494) were obtained from BD Pharmingen. Antibodies to phosphor-H2A.X (ser139) (07–164), acetyl-Histone H3 (06–599), acetyl-Histone H4 (06–866) and phosphor-Histone H3 (ser10) (05–1336), p53 (E26, 04–241) and cyclin B1 (05–373) were obtained from Millipore. Antibodies to survivin (sc-17779), β-actin (C4, sc-47778), goat anti-mouse IgG-HRP (sc-2005) and goat anti-rabbit IgG-HRP (sc-2004) were obtained from Santa Cruz Biotechnology, Inc. Antibody to TNF-α (ab9739) was obtained from abcam and antibodies AlexaFluor 488 goat anti-rabbit IgG (H+L) (A11008), AlexaFluor 594 goat anti-mouse IgG (H+L) (A11005) were obtained from invitrogen.

### Cellular Viability

Cells were seeded in triplicate in 96-well plates at a density of 5,000 cells/well into 96-well plates. After 24 h of culture, cells were treated with increasing concentrations of OME or equal volume of vehicle (ethanol) as control and incubated for the indicated time period. Cell viability was determined using a CellTiter-Glo Luminescent Cell Viability assay (Promega Corporation, Madison, USA), based on quantification of ATP, which signals the presence of metabolically active cells. Luminescent signal was measured using Berthold FB12 Luminometer. Data were presented as proportional viability (%) by comparing the treated group with the untreated cells, the viability of which is assumed to be 100%.

### Measurement of Caspase 3/7, 8 and 9 Activities

MDA-MB-231 cells were seeded at the density of 5,000 cells/well into 96-well plate in triplicate and treated with indicated concentrations of OME or equal volume of vehicle (ethanol) as control for 24 and 48. Caspase-3/7, 8 and 9 activities were measured using a luminescent caspase-Glo 3/7, caspase8 and caspase 9 assay kit (Promega Corporation, Madison, USA) following the manufacturer’s instructions. Briefly, caspase reagents were added to triplicate wells of a 96-well plate which was then mixed in an orbital shaker and incubated for 2.5 h at room temperature in the dark. Luminescent signal was measured as described above.

### Flow Cytometric Analysis of Cell Cycle

MDA-MB 231 cells were seeded in 100 mm culture dishes and cultured for 24 h before addition of various concentrations of OME or equal volume of vehicle (ethanol) as control. After incubation for the indicated time, cells were harvested by trypsin release, washed twice with ice-cold PBS, resuspended in 500 µl PBS, fixed with an equal volume of 100% ethanol and incubated for at least 12 h at −20°C. Before flow cytometry analysis, cells were pelleted, washed twice with PBS, permeabilized in 0.1% Triton X-100/PBS for 15 min on ice, pelleted and then resuspended in PBS containing 40 µg/ml propidium iodide and 25 µg/ml RNase A, and incubated at 37°C for 30 min. Cell samples were analyzed on the BD FACSCanto II (Becton Dickinson). Data acquisition was performed using FACSDiva 6.1 software. Percentage of cells in G1, S and G2/M phases were determined using the FlowJo software.

### Immunofluorescence Staining

MDA-MB 231 cells (3×10^4^) were grown in complete media on 4 well labtek chamber slide (Nunc) for 24 h, then treated with the indicated concentrations of OME or equal volume of vehicle (ethanol) as control for 24 h. Cells were then fixed in 10% formalin solution (4% paraformaldehyde) (Sigma-Aldrich) for 5 min at RT followed by permeabilization in PBS containing 0.1% Triton X-100 for 5 min at RT. Cells were then washed three times with PBS, blocked with 5% nonfat dry milk in PBS for 30 min at RT and incubated with the primary antibody diluted, at the concentration suggested by the manufacturer, in 1% nonfat dry milk/PBS overnight at 4°C. Following overnight incubation, cells were washed three times with PBS and placed for 1 h at RT in the presence of rhodamine-conjugated or fluorescein-conjugated secondary antibody diluted at 1∶200 in 1% nonfat dry milk/PBS. After washing with PBS, sample cells were mounted in Fluoroschield with DAPI (Sigma-Aldrich) and examined under Nikon Ti U fluorescence microscope.

### Cell Extract and Western Blotting Analysis

Cells (1.8×10^6^) were seeded in 100 mm culture dishes and cultured for 24 h before addition of various concentrations of OME or equal volume of vehicle (ethanol) as control. After incubation for the indicated times, cells were washed twice with ice-cold PBS, released by scrapping, pelleted and lysed in RIPA buffer supplemented with protease/phosphatase inhibitor cocktail. Following incubation for 30 min on ice, the cell lysate was obtained by centrifugation at 14,000 rpm for 20 min at 4°C. Protein concentration of lysates was determined by BCA protein assay kit (23225, Thermo Scientific) and the lysates were adjusted with lysis buffer. Aliquots of 25 µg of total proteins were resolved onto 10–12% SDS-PAGE. Proteins were transferred to nitrocellulose membranes (88018, Thermo Scientific) and blocked for 1 h at room temperature with 5% non-fat dry milk in TBST (TBS and 0.05% Tween 20). Incubation with specific primary antibodies was performed in blocking buffer overnight at 4°C. Horseradish peroxidise-conjugated anti-IgG was used as secondary antibody. Immunoreactive bands were detected by ECL chemiluminescent substrate (32209, Thermo Scientific). Membrane stripping by incubating the membrane in Restore western blot stripping buffer (21059, Thermo Scientific) according to the manufacturer’s instructions.

### Colony Formation Assay in Soft Agar

Assays were performed in six-well plates. The lower (base) layer consisted of 1 ml 2.4% Noble agar. The base layer was overlaid with a second layer consisted of 2.9 ml growth medium, 0.3% Noble agar, and 3×10^4^ MDA-MB-231 cells. Agar at 50°C was mixed with medium at 37°C, plated, and left to set for 10 min. Then 2 ml of growth medium was added on the top of the second layer. Plates were incubated at 37°C, humidified, and 5% CO_2_. Cells were allowed to grow in absence of treatment for 14 days until visible colonies were formed. Plates were fed twice a week with 2 ml of regular complete growth medium (DMEM). After 2 weeks, plates were fed with medium containing either the indicated concentrations of OME or equal volume of vehicle (ethanol) as control and the colonies were allowed to grow for one more week. Following treatment, the plates were washed twice with PBS and then colonies were fixed with 10% ice-cold methanol for 10 min and washed once with PBS. The colonies were allowed to stain for 1 hr in solution containing 2% Giemsa. The size of the colonies were measured, counted using a microscope (10X) and the colony size was categorized as Large (>200 µM) or small (50–200 µM). Colony sizes were expressed as a percentage of total counted colonies and then compared to the vehicle treated controls (ethanol). The experiment was repeated two times.

### Statistical Analysis

Results were expressed as means ± S.E.M. of the number of experiments. A Student’s *t*-test for paired or unpaired values was performed and a *p* value of <0.05 was considered statistically significant.

## Results

### *O. majorana* Extract Inhibits the Viability of the MDA-MB-231 Breast Cancer Cells

**Figure 1:**
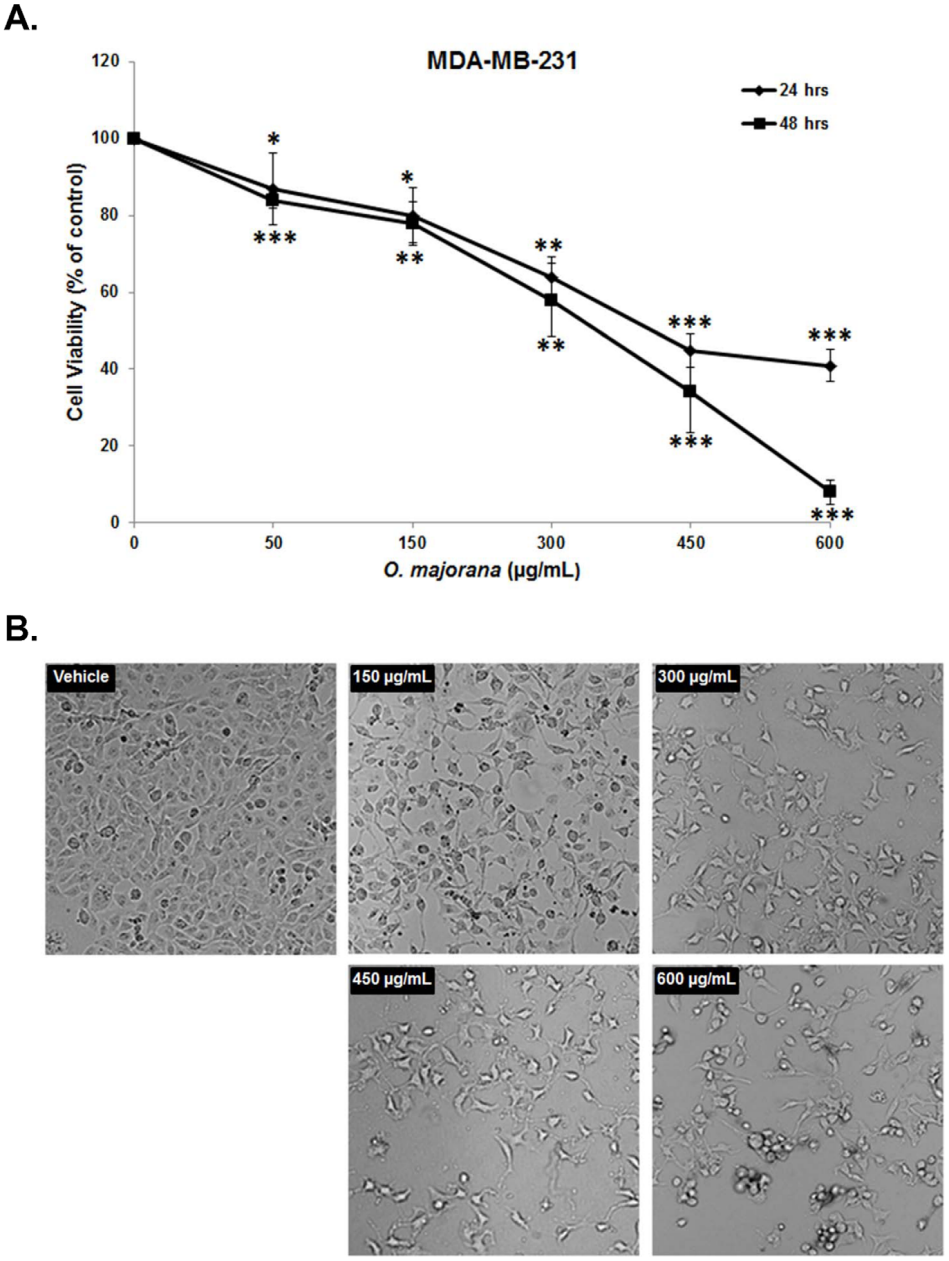
Effect of *O. majorana* extract on cell morphology and viability of human breast cancer cell MDA-MB-231 cells. Cells (5×10^3^ cells/well; 96-well plates) were cultured in DMEM supplemented with 10% Fetal bovine serum. (A) Cell viability (in percent) determined by using CellTiter-Glo Luminescent Cell Viability assay, after incubation of the exponentially growing cells with the indicated concentrations of OME or equal volume of vehicle (ethanol) as control for 24 and 48 h. (B) Micrograph of MDA-MB-231 cells, after 24 h incubation with various concentrations of OME. Data represent the mean of at least three independent experiments carried out in triplicate. Student’s t test was performed to determine the significance (*p<0.05, **p<0.005 and ***p<0.0005).

To examine the anticancer activity of *Origanum majorana* extract (OME) on breast cancer cells, we first measured the effect of various concentrations of the extract (0, 50, 150, 300, 450 and 600 µg/mL) on the proliferation of the MDA-MB-231 breast cancer cell line (Figure 1A). Our results show that the exposure of the MDA-MB-231 to OME decreased cellular viability in a concentration- and a time-dependent manner. The IC_50_ (producing half-maximal inhibition) was approximately 350 µg/mL at 24 h and 400 µg/mL at 48 h treatment. The observation of the OME-treated MDA-MB231 cells under light microscopy also revealed that the number of cells decreased when the concentration increased**.** Furthermore, as shown in figure 1B, light microscopy observation of MDA-MB231 cells treated with concentrations of 150, 300 and 450 and 600 µg/mL OME, underwent morphological changes characterized by a loss of their epithelial morphology visible after 24 h of treatment. Echinoid spikes and cellular rounding, characteristics of apoptotic cells were also observed.

### *O. majorana* Extract Leads to Mitotic Arrest and Apoptosis in MDA-MB-231 Cells

**Figure 2:**
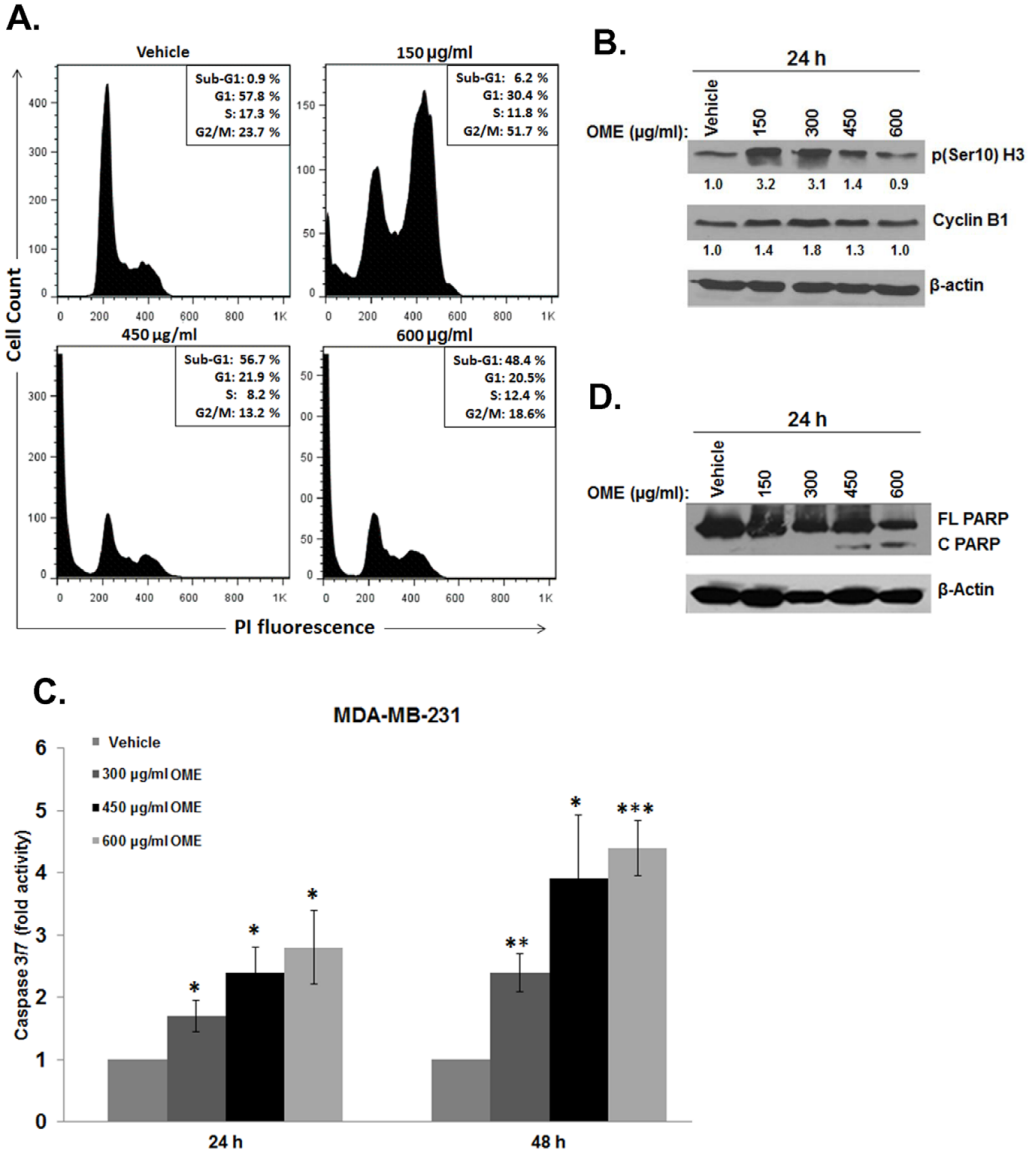
Induction of G2/M cell cycle arrest and apoptosis by *O. majorana* extract in MDA-MB-231 cells. (A) MDA-MB-231 cells (1.8×10^6^) seeded on 100 mm culture dish were exposed various concentrations of *O. majorana* extract or equal volume of vehicle (ethanol) as control for 24 h. Following treatment, cells were harvested, fixed, stained with propidium iodide, and analyzed for cell cycle distribution by flow cytometry. Data represent the mean of three independent experiments. The percentage of cells in sub-G1 (apoptosis), G1, S and G2/M appears at the upper right of each graph. (B) Expression of cell cycle regulator in OME-treated MDA-MB-231. Western blot analysis of phospho(ser10)-H3, and cyclin B1 in MDA-MB231 cells exposed for 24 h to ethanol or indicated concentrations of OME. (C) Stimulation of caspase 3/7 activity in MDA-MB-231 cells after exposure to OME (0–600 µg/mL) for 24 h and 48 h, relative to a similar amount of viable ethanol-treated cells. The relative caspase 3/7 activity was normalized to the number of viable cells per well and is expressed as fold of induction compared to the control. (D) Concentration-dependent induction of PARP cleavage in OME-treated MDA-MB231 cells. Cells were treated with or without increasing concentrations of the extract and proteins were extracted as described in Materials and Methods. Western blot analysis was carried out using anti-PARP antibodies. (*p<0.05, **p<0.005 and ***p<0.0005).

The ability of an anticancer drug to affect cell cycle distribution can provide information regarding its cytotoxic mechanism(s) of action. For this reason, we investigated the effect of OME on cell cycle distribution by flow cytometry. MDA-MB 231 cells were treated with indicated concentrations of *O. majorana* for 24 h and subjected to cell cycle analysis. At the concentration of 150 µg/mL, OME caused obvious G/2M arrest on these cells (figure 2A). Indeed, the population of G2/M increased significantly from 23 to 51.7% as the concentration of the OME increased to 150 µg/mL, indicating that OME-treated MDA-MB-231 cells were arrested in G2/M phase. A slight increase in the sub-G1 population (6.2%) indicates that a small population of OME-treated MDA-MB-231 cells are undergoing cell death was also observed by these concentrations. Similar results were obtained for 300 µg/mL of OME (data not shown). Interestingly, at higher concentrations of OME, flow cytometry analysis revealed a dramatic increase in the apoptotic population (sub-G1 peak) raising from 0.9% in the control to 48.4% and 56.7% in cells treated with 600 and 450 µg/mL, respectively (Figure 2A).

To determine whether OME induced cell cycle arrest specifically at mitosis or G2 phase, we examined the phosphorylation status of histone H3 (Ser 10). Histone H3 is phosphorylated at serine 10 during mitosis by aurora kinase and the phosphorylation status of H3 is considered a marker of mitosis [21]. We therefore, investigated the expression of p(ser10)H3 and found that treatments 150 and 300 µg/mL of *O. majorana* significantly increase the phosphorylation level of histone H3 (Figure 2B, upper panel). This result indicates that OME induces mitotic arrest of MDA-MB231 cells. Next, we investigated the mechanism of OME-induced mitotic arrest. Accumulation of cyclin B1 is well known to play an important role in G2/M transition. Knock-down of cyclin B1 with siRNA produces cell cycle arrest predominantly in the G2 phase [22], while an upregulation of cyclin B1 invokes mitotic arrest [23]. We, therefore, investigated the protein level of cyclin B1 in OME-treated MDA-MB-231 cells. We found that treatment with these concentrations of OME leading to mitotic arrest, caused also an increase of cyclin B1 protein in MDA-MB-231 cells (Figure 2B, lowe panel), suggesting that cyclin B1 accumulation might play a crucial role in OM triggered mitotic arrest. Taken together, our data reveal a differential concentration effect of OME on cell cycle progression of the MDA-MB 231 cells. Whereas, lower concentrations of OME (150 and 300 µg/mL) induced a major mitotic arrest with a slight increase in the apoptotic population, higher concentrations (450 and 600 µg/mL) induced a massive cell death by apoptosis. The reduced cell viability observed in MDA-MB231 cells treated with low concentrations of OME, is possibly due to a large extent to an inhibition of cell proliferation rather than to cell death.

Apoptosis in OME-treated MDA-MB-231 cells was further examined by measuring caspase 3/7 activation in MDA-MB 231 cells treated with various concentrations (300, 450 and 600 µg/mL) of OME after 24 h of treatment. A concentration- and time-dependent activation of caspase 3/7 was detected in treated cells (Figure 2C). Interestingly, cleavage of the poly(ADP-ribose) polymerase (PARP) occurred only in cells treated with higher (450 and 600 µg/mL) but not at lower concentrations (150 and 300 µg/ml) of OME (Figure 2D). It is noteworthy to mention that at these lower concentrations of OME, only low apoptotic induction rate was observed despite the detection of caspase 3/7 activation.

### Concentration-dependent Regulation of Survivin Expression by *O. majorana* Extract

**Figure 3:**
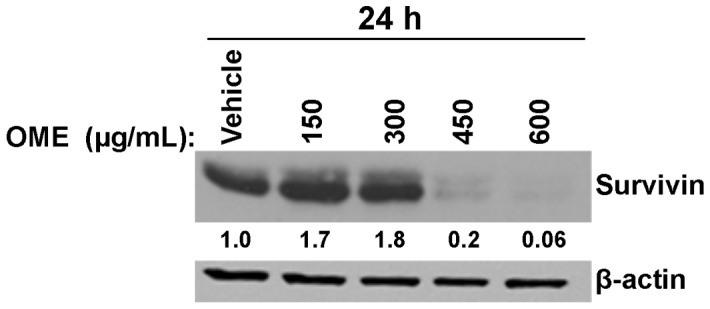
Differential regulation of survivin expression by *OME* in MDA-MB-231 cells. Western blot analysis showing a differential effect on survivin expression by different concentrations of OME in MDA-MB-231 cells. Whole cell protein were extracted from OME or vehicle (ethanol)-treated cells and subjected to Western blot analysis, as described in Materials and Methods, for survivin and β-actin (loading control) proteins.

Survivin, a member of the inhibitor of apoptosis protein (IAP) family, plays an important role in both the regulation of cell cycle and the inhibition of apoptosis. Survivin levels increase in G2/M phase conferring resistance to apoptosis to the G2/M arrested cells. However, a decrease in survivin levels sensitizes the cells to apoptosis. Several studies have reported that survivin exerts its negative effect on apoptosis by inhibiting the activity of caspase 3, 7 and 9. Therefore, we examined a possible involvement of survivin in the cell cyle arrest and apoptosis triggered by OME. Toward this, we have analyzed, by Western blotting, the expression of survivin in response to various concentrations of OME after 24 h treatment. Interestingly, we observed a differential concentration-effect of OME on survivin expression on the MDA-MB-231 cells (Figure 3). We found that low concentrations of OME led to a substantial increase in the level of survivin, while higher concentrations caused a drastic decrease of survivin (99%). Based on these results, we conclude that OME exerts a concentration-dependent effect on MDA-MB-231 cells. Low concentrations of OME induced a mitotically arrested cells accompanied by survivin upregulation which, in turn, conferred resistance to cell death to this population of cells, probably by inhibiting the activity of caspase 3/7 which was monitored by the absence of PARP cleavage at these concentrations. Treatment of MDA-MB-231 cells with higher concentrations of OME caused a dramatic decrease in survivin expression and consequently sensitized MD-MB-231 cells to apoptosis.

### *O. majorana* Extract Activates the Extrinsic Pathway for Apoptosis via an Upregulation of TNF-α and Activation of Caspase 8

**Figure 4:**
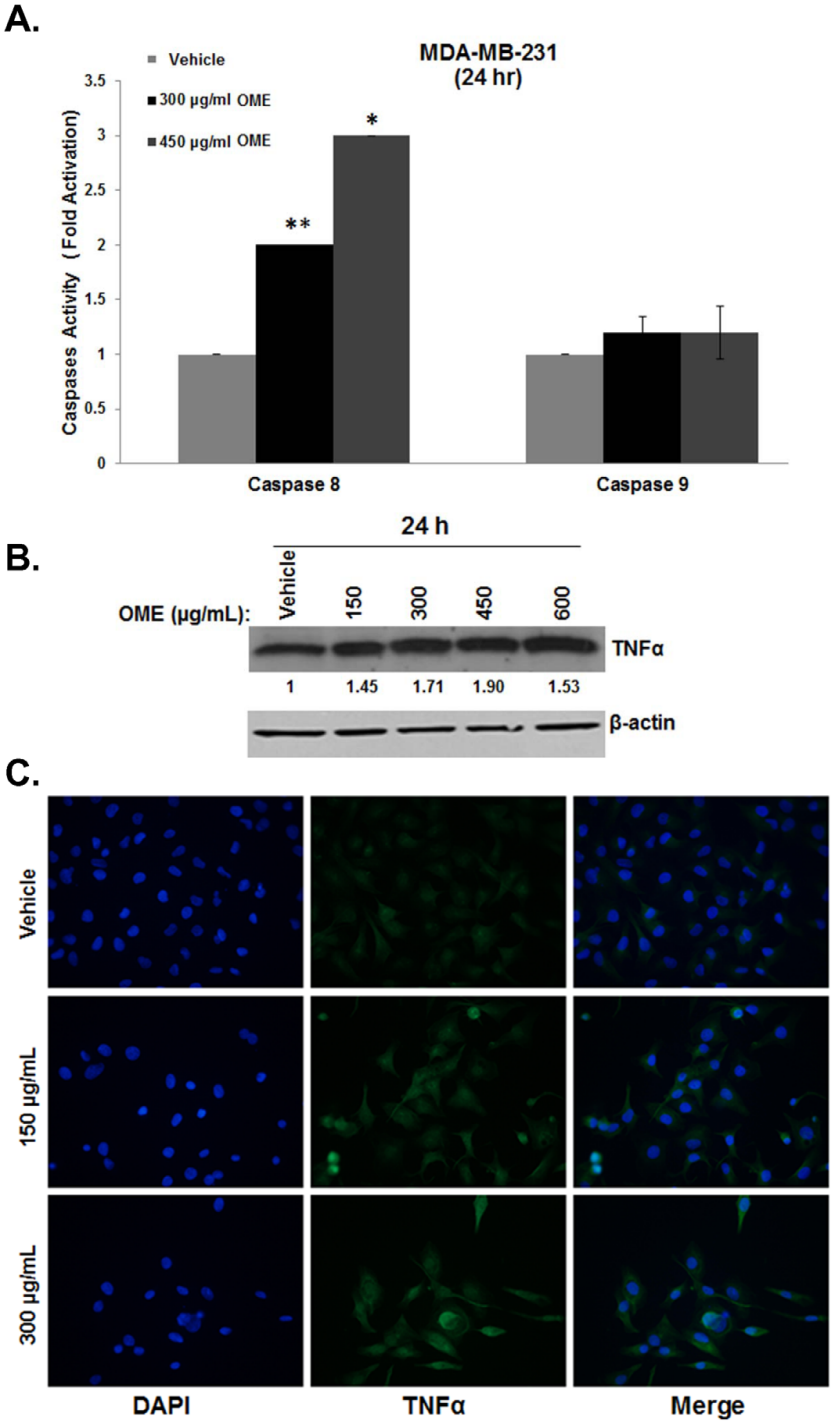
*O. majorana* induces apoptosis by activation of caspase 8 and upregulation of TNF-α. (A) *O. majorana* extract induces an activation of caspase 8 but not caspase 9 in MDA-MB-231 cells. MDA-MB-231 cells were incubated with various concentrations of the extract for 24 h. The caspase activation induced by the OME was assayed as described under Materials and methods. The relative caspase 8 and 9 activity was normalized to the number of viable cells per well and is expressed as fold of induction compared to the control. (B) Western blot analysis showing an increase in cellular TNF-α protein in the MDA-MB-231 cells treated with OME. Whole cell protein were extracted from OME-treated cells or vehicle (ethanol)-treated cells and subjected to Western blot analysis, as described in Materials and Methods, for TNF-α and β-actin (loading control) proteins. (C) Immunofluorescence staining for TNF-α in OME-treated MDA-MB- 231cells. Cells were treated with 150 and 300 µg/mL of the extract for 24 h, fixed, permeabilized, and then processed for immunofluorescence using antibodies against TNF-α protein. DAPI was used as a nuclear stain. (*p<0.05 and **p<0.005).

Having shown that OME induces the activation of the effector caspases 3/7, we looked at the activity of the initiator caspases of the extrinsic and intrinsic cell death pathway, namely caspase 8 and caspase 9, respectively. Surprisingly, no caspase 9 activation was detected in response to various concentrations of OME after 24 h of treatment (Figure 4A). On the other hand, caspase 8 activity increased in a concentration-dependent manner in response to OME treatment (Figure 4A). This result demonstrates that the apoptotic effect of the extract on MDA-MB-231 is dependent on caspase 8 activity, which implicates only the extrinsic cell death pathway since neither caspase 9 activation (Figure 4A) nor a change in Bax/Bcl2 ratio (data shown) were observed. After showing that the extrinsic cell death pathway is implicated in OME-dependent apoptosis, we were then interested in determining how this pathway is activated by OME. We determined the changes in the expression level of the tumor necrosis factor alpha (TNF-α) in response to OME after 24 h treatment. Western blot analysis revealed a clear increase in the level of TNFα in MDA-MB-231 cells in response to OME treatment (Figure 4B). The upregulation of TNF-α was further confirmed by immunofluorescence assay (Figure 4C). Even though we have shown that OME exerts its effect via the activation of the extrinsic pathway of cell death, we cannot rule out, at this stage, the possibility of OME-dependent apoptosis could also be triggered by another mechanism.

### ***O. majorana*** Lead to Depletion of Mutant p53 in MDA-MB-231 and Upregulation of p21^WAF1/CIP1^

**Figure 5:**
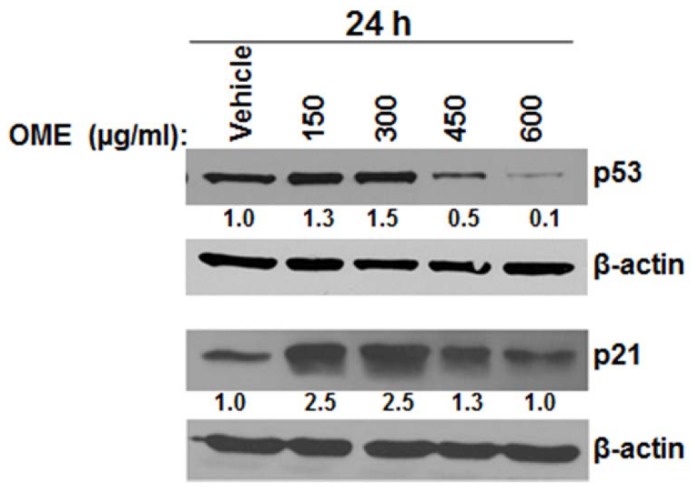
Expression levels of mutant p53 and p21 in *O. majorana*-treated MDA-MB-231cells. Cells were treated with various concentrations of (150, 300, 450 and 600 µg/mL) of OME or equal volume of vehicle (ethanol) as control for 24h and the expression of mutant p53, p21 and β-actin were estimated by Western blot.

Next, we tested the effect of OME on the expression of the tumor suppressor p53 in MDA-MB-231. Toward this aim, cells were treated with various concentrations of OME and the protein level of the mutant p53 determined. We found that low concentrations of 150 and 300 µg/mL of OME led to a slight increase in the protein level of mutant p53 (Figure 5, upper pannel). Most importantly, Western blotting analysis revealed apoptotic concentrations (450 and 600 µg/mL of OME) led to almost complete depletion of mutant p53 in MDA-MB-231 cells. This result is a potentially important finding because of the role of mutant p53 protein in human cancers. Because mutant p53 renders cancer cells more resistant to anticancer drugs, abolishing mutant p53 may therefore offer a promising approach for cancer prevention and therapy.

Because p21 protein has been reported to inhibit growth and apoptosis, we investigated whether the growth inhibition mediated by low concentrations (150 and 300 µg/mL of OME) was also associated with an induction of p21. Western blotting showed an upregulation of p21 protein with at least 2.5 fold increase in cells treated with low concentrations of OME, while a little or no effect on p21 expression was observed with higher concentrations of OME (Figure 5, lower panel). Based on that, we can postulate that p21 upregulation contributes, at least partially, to the cell cycle arrest observed with lower concentrations, while it has little or no role in cell death occurring at higher concentrations of OME.

### *O. majorana* Extract Induces Hyperacetylation of Histone H3 and H4 in the MDA-MB 231 Cells

**Figure 6:**
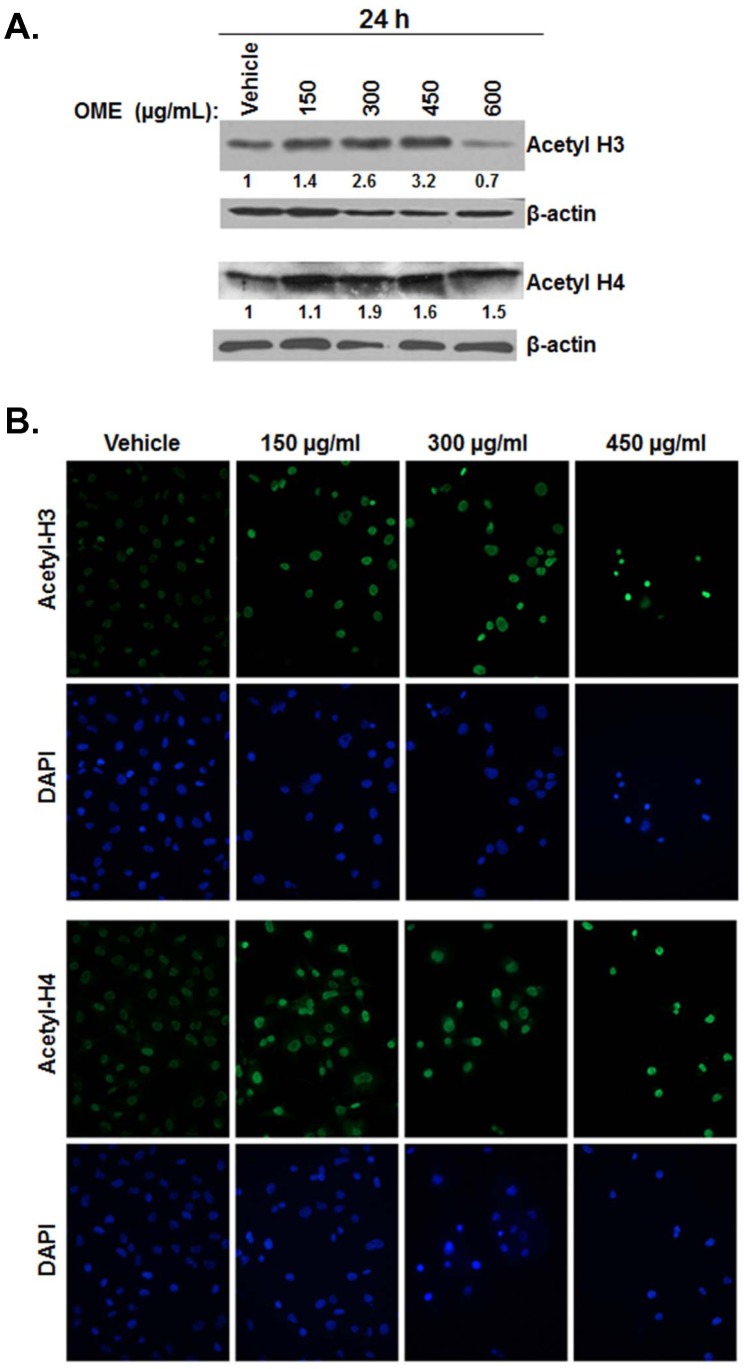
*O. majorana* induces hyperacetylation of histone H3 (AcH3) and H4 (AcH4). (A) Protein levels of Ac-H3 and Ac-H4, extracted from OME-treated cells, were detected by western blot using antibodies specific for the modified histone (Ac-H3) and Ac-H4. β-actin was used as loading control. (B) Immunofluorescence staining of Ac-H3 and Ac-H4 in MDA-MB231 cells treated with 150, 300 and 450 µg/mL of OME or equal volume of vehicle (ethanol) as control for 24 h. Cells were fixed, permeabilized, and then processed for immunofluorescence using antibodies against the indicated modified histones. DAPI was used as a nuclear stain.

Previously, expression of p21 and increased histone hyperacetylation have been linked to apoptosis and to growth arrest. Therefore, we examined the acetylation profile of histone H3 and H4 in MDA-MB-231 in response to treatment for 24 h to increasing concentrations of OME. As shown in figure 6A, the time course analysis showed a gradual increase in acetylated histones, H3 and H4. A marked overall increase in the acetylation status of histone H3 and H4 was also detected by immunofluorescence staining (Figure 6B). Altogether, these results showed that OME induced hyperacetylation of histone H3 and H4.

### Accumulation γH2AX, a Marker of Double Strand Breaks, in *O. majorana* Extract Treated MDA-MB 231 Cells

**Figure 7:**
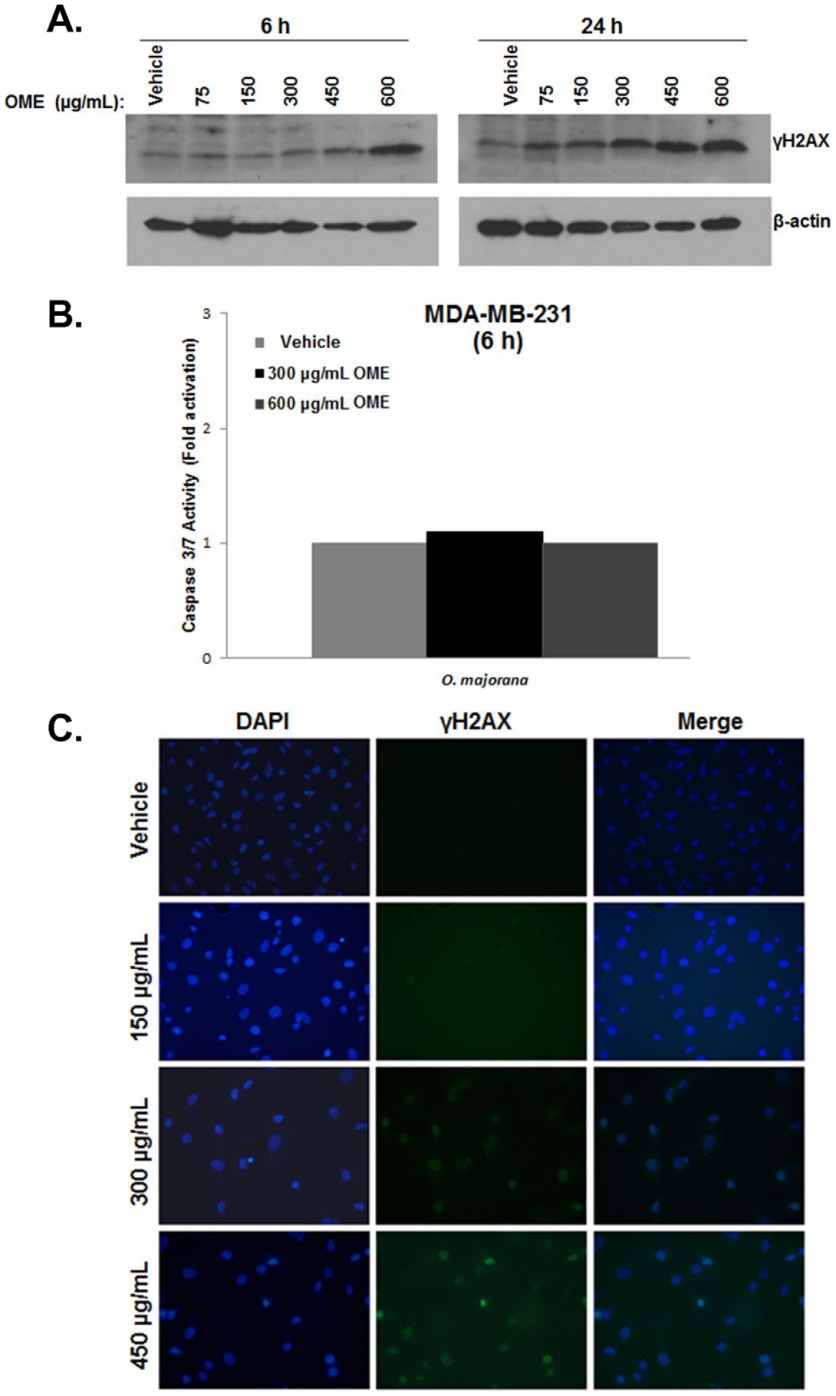
*O. majorana* extract induces a dose-dependent activation of γH2AX, a marker of DNA double-strands breaks, in MDA-MB 231 cells. (A) Western blot analysis of phosphor-H2AX (ser 139) in MDA-MB231 cells exposed for 6 and 24h with the indicated concentrations of OME or equal volume of vehicle (ethanol) as control. (B) Immunofluorescence staining for γH2AX in OME-treated MDA-MB 231cells. Cells were treated with vehicle or 150, 300 and 450 µg/mL extract for 24 h, fixed, permeabilized, and then processed for immunofluorescence using antibodies against p-H2AX (ser 139) protein. DAPI was used as a nuclear stain.

We sought to investigate whether OME induced DNA damage in MDA-MB-231 cells. For this purpose, MDA-MB 231 cells were cultured for 6 and 24 h in complete media containing either ethanol (control) or increasing concentrations of OME (75, 150, 300, 450 and 600 µg/mL). DNA damage was determined by measuring the levels of phosphorylated H2AX (γH2AX) after 6 and 24 h of treatment of the MDA-MB-231 cells with OME. Western blotting analysis revealed a time- and a concentration-dependent increase in the levels of γH2AX in response to OME treatment (Figure 7A), indicating an accumulation of double strand breaks in these cells. The increase in DNA damage was also assessed by immunofluorescence staining of γH2AX in cells treated with 150, 300 and 450 µg/mL OME for 24 h. Figure 7C clearly shows a concentration-dependent increase of γH2AX foci in response to OME. Since the activation of γH2AX occured as early as 6 h, a time in which no cell death (data not shown) or caspase 3/7 activation were observed (Figure 7B), this rules out the possibility that the resulting DNA damage is a consequence of DNA fragmentation resulting from caspases' activities and further confirms the potential of this OME extract to induce double strand DNA breaks in a dose-dependent manner.

### *O. majorana* Inhibits Colony Growth of MDA-MB-231

**Figure 8:**
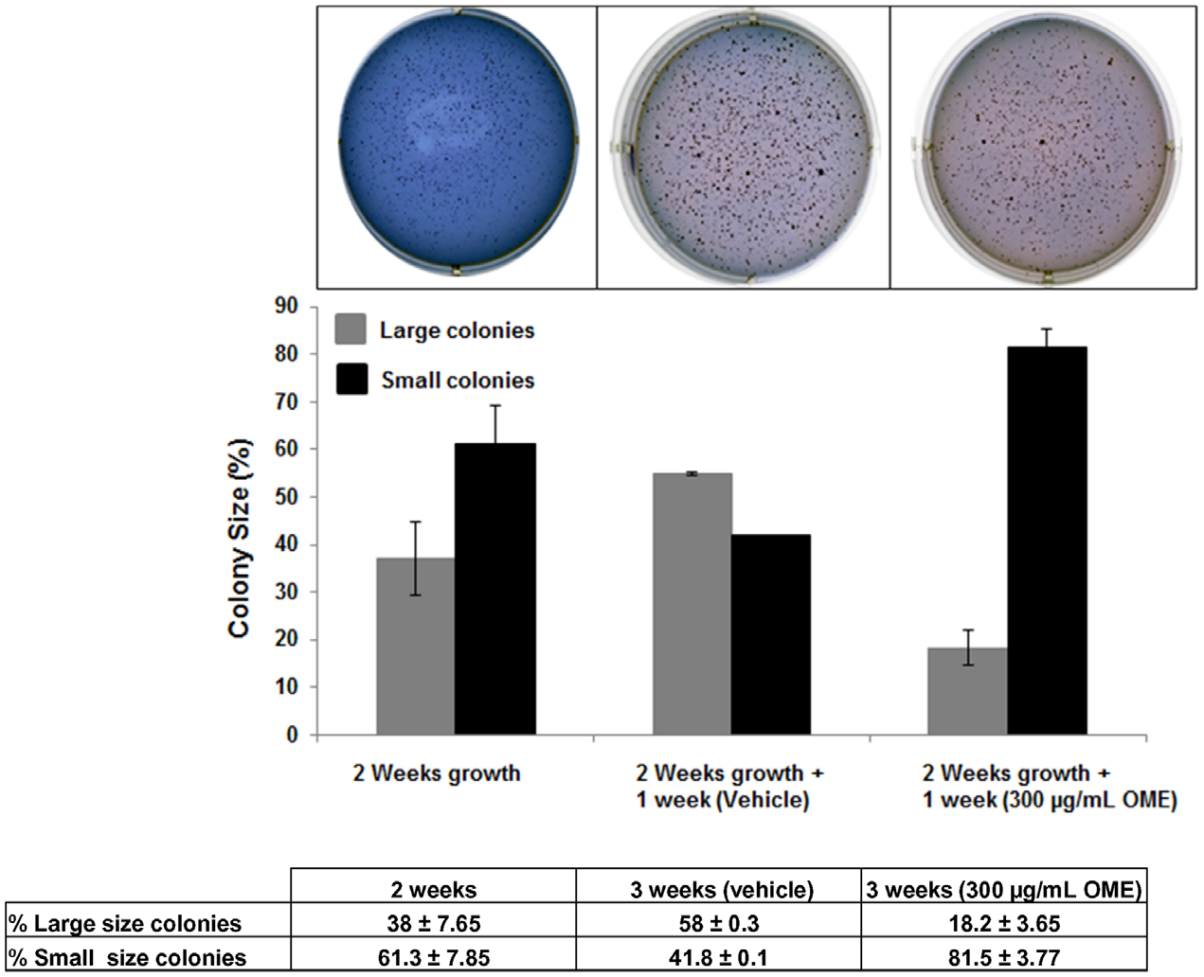
Inhibition of colony growth by *O. majorana* extract. Inhibition of colony growth was assessed by measuring the size of the colonies obtained in vehicle (ethanol)- and OME-treated plates. Data were compared with those obtained for the 2 weeks colonies. Two types of colonies were counted and depending on their diameter were categorized as large (≥200 µm) and small (<200−≥50 µm).

To further confirm the inhibitory potential of *O. majorana* on MDA-MB 231 cells, we sought to determine if OME could inhibit the further growth of already formed MDA-MB-231 colonies. For this purpose, MDA-MB-231 cells were first allowed to grow and form visible colonies in absence of treatment. After 14 days of growth, colonies were incubated with ethanol as control and with OME and allowed to grow for one more week. Figures 8 shows that the size of the ethanol-treated (control) colonies kept growing compared to the size of the two weeks colonies; more large colonies were obtained in the three weeks plate, while less small colonies were counted, indicating that small colonies became larger in size. Interestingly, OM-treated colonies shows regression in colony size compared to the two weeks colonies. In OM-treated plates, the number of large size colonies counted was less than what was obtained in the two weeks plate, while the number of small colonies was significantly greater, suggesting size regression in the large colony induced by OME. This result along with the viability and flow cytometry data confirm the anti-cancer effect of OME on the triple negative mutant p53 MDA-MB-231 breast cancer cells.

## Discussion

Common cancer treatment drugs aim at inhibiting the cell cycle progression and at inducing cell death and apoptosis. Cancer chemoprevention through these two events (cell cycle arrest and apoptosis) has been reported for several natural compounds [6]–[10].

In the present study, we have shown that the extract of an ethanolic fraction of *Origanum majorana* inhibited the proliferation of the mutant p53 triple negative breast cancer (TNBC) cell line, MDA-MB-231. We have also demonstrated that OME induces a differential concentration-dependent effect on these cells. OME induces a cell cycle arrest at G2/M phase and more precisely a mitotic arrest at low concentrations. This finding is supported by a body of evidence: (*i*) increase in the level of p(ser10)H3, a mitotic marker, and (*ii*) an increase of cyclin B1 protein level, whose upregulation was reported in several mitotically arrested cells. We have also shown that the cell cycle arrest correlates with an upregulation of the CDK inhibitor p21 and the anti-apoptotic protein, survivin. At high concentrations, however, OME induced a massive apoptosis demonstrated by a dramatic increase in the sub-G1 population. We have demonstrated that OME exerts its apoptotic effect by activating the cell death extrinsic pathway is mediated, at least partially, via the activation of TNF-α. We have also shown that OME-induced apoptosis is also mediated by an increase in DNA damage, revealed by an upregulation of γH2AX, severe depletion of the mutant p53 and survivin proteins from the treated cells.

The process of apoptosis can be induced either by the extrinsic pathway which involves signalling from death receptors at the cell surface or by the intrinsic mitochondria-mediated pathway [24]. Activation of the death receptor-mediated apoptosis requires the interaction of the ligands such as TNF-α and Fas with their transmembrane receptors [25]. The ligand-receptor interaction leads to the activation of the effector caspase 8, which in turn activates the effector caspase 3 directly and/or through mitochondria [26]. The mitochondria-mediated apoptosis pathway is associated with permeabilization of the mitochondria outer membrane, reduced mitochondrial membrane potential (Δ*ψ*m), change in expression of the anti-apoptotic Bcl2 family members, such as Bcl2 and Bcl-XL and the pro-apoptotic members such as Bax and Bak leading to the formation of the apoptosome, activation of caspase 9 and consequently activation of caspase 3 [27]. Intrinsic- and extrinsic-pathaways activated caspase 3 cleave poly(ADP-ribose) polymerase (PARP), thus resulting in apoptosis [28]. In the present study, we showed that OME induced TNF-α-mediated extrinsic apoptotic pathway. TNF-α receptor was activated at 24 h and induced downstream signalling such as caspase 8, caspase 3, and PARP cleavage. On the other hand, OME did not induce mitochondria-mediated apoptotic pathway since no change in the BAX/Bcl2 ratio or activation of caspase 9 were detected. We suggest that OME induces apoptosis solely through TNF-α activated signal pathway in MDA-MB-231 cells.

Studies have reported that DNA damage is one of the molecular events associated with cell cycle arrest and apoptosis. Indeed, and many anti-cancer drugs have been shown to induce DNA damage [29], [30]. Moreover, cancer cells are reported to be more susceptible than normal cells to DNA damaging agents [31], therefore there is a growing interest in dietary phytochemicals that possess DNA-damaging activity. In the present study we showed that OME elicited DNA damage measured by an increase in a concentration-dependent manner of the marker of DNA damage, γ-H2AX, after treatment with OME for 6 or 24 h. The differential response to the different concentrations of OME (G2/M arrest and/or apoptosis), may be partially mediated by the extent of DNA damage occurred within the genome. Low levels of DNA damage may trigger recruitment of DNA repair complexes, expression of anti-apoptotic and survival proteins leading to arrested cell cycle until the genotoxic lesions are repaired. In this case, survival protein such as survivin gets activated in order to maintain the viability of G2/M arrested cells. On the other hand, when genomes are overwhelmed by DNA damage, cells are eliminated by apoptosis [32], [33]. In this study, we have found that high concentrations of OME triggered high level of DNA damage to the genome, causing cell to enter apoptosis. Our data suggest that *Origanum majorana* possess a genotoxic effect on MDA-MB-231 cells. At this stage, the mechanism(s) by which OME induces DNA damage remain(s) unknown, and certainly deserves further studies.

Inhibitor of apoptosis proteins (IAPs), which includes survivin, represents a family of anti-apoptotic proteins that bind and inactivate active caspase 3, 7 [34], [35] and caspase 9 [36] and can modulate cell division and cell cycle progression [37].

Interestingly, survivin has no effect on caspase 8 activity. Survivin has been shown to be highly expressed in most cancers, where it functions as inhibitor of apoptosis. In breast cancer, overexpressed survivin was shown to protect cells against apoptosis induced by chemotherapeutic agents, such as etoposide [34]. Based on these reports, survivin protein represents an attractive target of particular importance in cancer therapy at large and in breast cancer therapy in particular. In consideration of the recognized role for survivin as a custodian of cancer cell survival, our results suggest that OME might exert its cytotoxic anti-cancer effects at least partly via the down-regulation of survivin. In our study, we have shown that survivin expression is differentially regulated in a concentration-dependent manner by OME. Lower concentrations of OME induced an upregulation of survivin which causes cells to arrest the cell division and to resist apoptosis by inhibiting the cell death program. In fact, we showed that in these arrested cells, PARP cleavage was not detected despite the activation of caspase 3/7. This effect might be mediated by the inhibition of active 3/7 by the upregulated survivin. Survivn function could also account for the mitotic arrest induced by OME. In fact, Survivin, has also been shown to be required for mitotic arrest of Hela cells induced by the anticancer drug UCN-01 [38].

The tumor suppressor protein, p53 is found to be mutated in about 50% of human cancers [39]. Mutant p53 is reported to play a key role in cancer cells resistance to certain anticancer drugs and thus is considered as a potential cancer-specific target for pharmacologic interventions [40], [41]. Studies have shown that inhibition of mutant p53 by RNA interference sensitizes cancer cell to cell death by chemotherapeutic agents [42]. Wang et al. 2011, showed that the naturally occurring isothiocyanates (ITCs) phenetyl isoisothiocyanate (PEITC), derived from watercress plant, and the synthetic ITC, 2,2-di phenetyl isoisothiocyanate selectively deplete mutant, but not the wild-type p53, and induce apoptosis in many cancer cells, including the MDA-MB-231 breast cancer cells [43]. Here, we showed that OME led to dramatic decrease in the mutant p53 level in MDA-MB-231 cells. As such, mutant p53 depletion may be an important target for chemoprevention and therapy by *O. majorana* for TNBC.

Increase in the expression of the cyclin-dependent kinase inhibitor, p21 has been shown to augment G2/M arrest via a p53-independent mechanism in human breast cancer [44]. In most cases, growth arrest was found to be associated with apoptosis. In this study, we showed that low concentrations of OME treatment led to G2/M arrest without significant increase in cell death after 24h treatment. Histone hyperacetylation has been demonstrated to be directly linked to the upregulation of p21 and this activation can also occur independently of p53 [45]. Moreover, histone hyperacetylation was also shown to be associated with growth suppression and apoptosis. Our data showed that OME induced histone H3 and H4 hyperacetylation in MDA-MB-231 cells, suggesting that the anti-breast cancer effects of OME were at least partly mediated by histone H3 and H4 hyperacetylation by regulating the expression of the genes controlling these two events. The mechanism by which OME induces histone hyperacetylation might involve a histone deacetylase inhibitor (HDI) activity. Interestingly, the plant, *O. majorana*, contains luteolin, a dietary flavonoid with HDI activity [14]. In fact, luteolin was able to decrease the viability of lung, colon, liver and breast cancer cells and induce hyperacetylation of histone H3 and H4 [46]. In light of these data, we conclude that the histone hyperacetylation induced by OME is involved with the HDI activity of luteolin. We are currently undertaking further investigations to better understand the mechanim(s) by which OME induces histone hyperacetylation.

**Figure 9:**
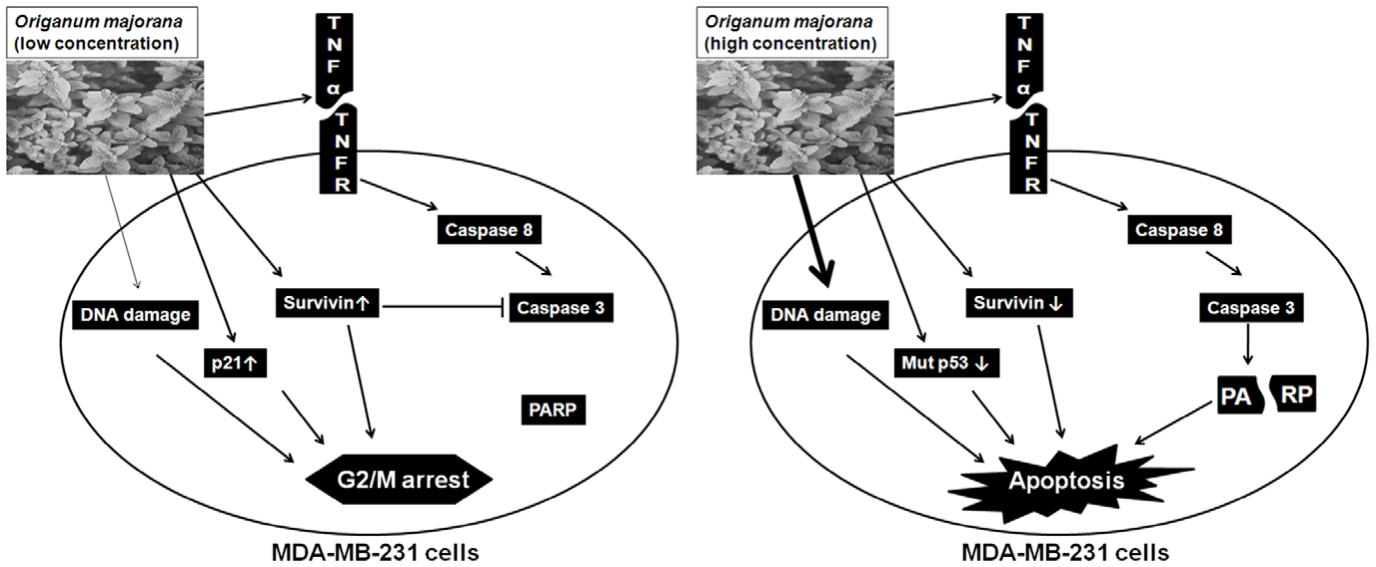
The proposed signal pathways on *O. majorana-*induced cell cycle arrest, at low concentrations, and apoptosis, at high concentrations, in the triple negative mutant p53 human breast cancer MDA-MB-231 cells.

In conclusion, our data are consistent with a model shown in figure 9 which shows the concentration-dependent differential effect of *O.majorana* extract on mutant p53, triple negative MDA-MB-231 cells. At low concentrations, OME induced a mitotic arrest associated with low level of DNA damage (Figure 9, thin arrow), upregulation of the CDK inhibitor p21 and the inhibitor of apoptosis, survivin. We believe that these events along with other “yet to be identified” events contribute to the cell cycle arrest. In addition we also propose that, at these concentrations, survivn is also implicated in the blockade of the TNF-mediated apoptosis pathway, by directly inhibiting the activity of the active caspase 3. On the other hands, high concentrations, OME induce massive apoptosis via the activation of the TNF-α extrinsic pathway which is associated with high level of DNA damage (Figure 9, thick arrow) and almost complete depletion of the mutant p53 and surviving proteins from these cells. Our findings provide the first instance of a potential **r**ole for OME as an anti-breast cancer agent *in vitro* which certainly deserves more attention for further explorations to identify novel compounds for breast cancer.

## References

[1] American Cancer Society. Cancer facts and figures (2012) Available: http://www.cancer.org/acs/groups/content/@epidemiologysurveilance/documents/document/acspc-031941.pdf.Accessed 2013, Jan 30.

[2] Cragg GM, Newman DJ (2000) Antineoplastic agents from natural sources: achievements and future directions. Expert Opin Investig Drugs 9: 2783–2797. 10.1517/13543784.9.12.2783 11093353

[3] Kaczirek K, Schindl M, Weinhausel A, Scheuba C, Passler C, et al (2004) Cytotoxic activity of camptothecin and paclitaxel in newly established continuous human medullary thyroiod carcinoma cell lines. J Clin Endocrinol Metabol 89: 2397–240. 10.1210/jc.2003-031314 15126569

[4] Nawab A, Yunus M, Mahdi AA, Gupta S (2011) Evaluation of Anticancer Properties of Medicinal Plants from the Indian Sub-Continent. Molecular and Cellular Pharmacology 3: 21–29.

[5] Watson WH, Cai J, Jones DP (2000) Diet and apoptosis. Annual Review of Nutrition 20: 485–505. 10.1146/annurev.nutr.20.1.485 10940343

[6] Kuno T, Tsukamoto T, Hara A, Tanaka T (2012) Cancer chemoprevention through the induction of apoptosis by natural compounds. Journal of Biophysical Chemistry 3: 156–173.

[7] Teiten M-H, Gaascht F, Eifes S, Dicato M, Diederich M (2010) Chemopreventive potential of curcumin in prostate cancer. Genes Nutr 5: 61–74. 19806380 10.1007/s12263-009-0152-3 PMC2820199

[8] Adams LS, Phung S, Yee N, Seeram NP, Li L, et al (2010) Blueberry Phytochemicals Inhibit Growth and Metastatic Potential of MDA-MB-231 Breast Cancer Cells through Modulation of the Phosphatidylinositol 3-Kinase Pathway. Cancer Reseach 70: 3594–3605. 10.1158/0008-5472.CAN-09-3565 PMC2862148 20388778

[9] Gu Y-H, Leonard J (2006) *In vitro* effects on proliferation, apoptosis and colony inhibition in ER-dependent and ER independent human breast cancer cells by selected mushroom species. Oncology Reports 15: 417–423. 16391863

[10] Lee HS, Seo EY, Kang NE, Kimb WK (2008) [6]-Gingerol inhibits metastasis of MDA-MB-231human breast cancer cells. Journal of Nutritional Biochemistry 19: 313–319. 17683926 10.1016/j.jnutbio.2007.05.008

[11] Vàgi E, Rapavi E, Hadolin M, Vàsàrhelyiné Perédi K, Balàzs A, et al (2005) Phenolic and triterpenoid antioxidants from Origanum majorana L. herb and extracts obtained with different solvents. Journal of Agricultural and Food Chemistry 53: 17–21. 15631502 10.1021/jf048777p

[12] Al-Harbi NO (2011) Effect of marjoram extract treatment on the cytological and biochemical changes induced by cyclophosphamide in mice. Journal of Medicinal Plants Research 5: 5479–5485.

[13] Miron TL, Plaza M, Bahrim G, Ibariez E, Herrero M (2011) Chemical composition of biocactive pressurized extracts of Romanian aromatic plants. Journal of Chromatography A. 1218: 4918–4927. 10.1016/j.chroma.2010.11.055 21163488

[14] Tsimogiannis D, Stavrakaki M, Oreopoulou V (2006) Isolation and characterisation of antioxidant components from Oregano (*Origanum heracleoticum*). InternationalJournal of Food, Science, and Technology 41: 39–48.

[15] Leeja L, Thoppil JE (2007) Antimicrobial activity of methanol extract of *Origanum majorana* L. (Sweet marjoram). J. Environ. Biol. 28: 145–146. 17718003

[16] El-Ashmawy IM, El-Nahas AF, Salama OM (2005) Protective effect of volatile oil, alcoholic and aqueous extracts of *Origanum majorana* on lead acetate toxicity in mice. Basic Clin. Pharmacol. Toxicol. 97: 238–243. 10.1111/j.1742-7843.2005.pto_136.x 16176560

[17] El-Ashmawy IM, Amal S, Salama OM (2007) Acute and long term safety evaluation of *Origanum majorana* essential oil. Alex. J. Pharm. Sci. 21: 29–35.

[18] El-Ashmawy IM, Saleh A, Salama OM (2007) Effects of marjoram volatile oil and grape seed extract on ethanol toxicity in male rats. Basic Clin. Pharmacol. Toxicol. 101: 320–327. 10.1111/j.1742-7835.2007.00125.x 17910615

[19] Yazdanparast R, Shahriyary L (2008) Comparative effects of *Artemisia dracunculus*, *Satureja hortensis* and *Origanum majorana* on inhibition of blood platelet adhesion, aggregation and secretion. Vascul. Pharmacol. 48: 32–37. 10.1016/j.vph.2007.11.003 18069068

[20] Lin LT, Liu LT, Chiang LC, Lin CC (2002) In vitro anti-hepatoma activity of fifteen natural medicines from Canada. Phytotherapy Research 16: 440–444. 12203264 10.1002/ptr.937

[21] Hendzel MJ, Wei Y, Mancini MA, Van Hooser A, Ranalli T, et al (1997) Mitosis-specific phosphorylation of histone H3 initiates primarily within pericentromeric heterochromatin during G2 and spreads in an ordered fashion coincident with mitotic chromosome condensation. Chromosoma 106: 348–60. 9362543 10.1007/s004120050256

[22] Maurer M, Komina O, Wesierska-Gadek J (2009) Roscovitine differentially affects asynchronously growing and synchronized human MCF-7 breast cancer cells. Ann N Y Acad Sci 1171: 250–256. 19723062 10.1111/j.1749-6632.2009.04717.x

[23] Choi HJ, Fukui M, Zhu BT (2011) Role of Cyclin B1/Cdc2 Up-Regulation in the Development of Mitotic Prometaphase Arrest in Human Breast Cancer Cells Treated with Nocodazole. PLoS ONE 6(8): e24312 doi:10.1371/journal.pone.0024312. 21918689 10.1371/journal.pone.0024312 PMC3168870

[24] Konopleva M, Zhao S, Xie Z, Segall H, Younes A, et al (1999) Apoptosis. Molecules and mechanisms. Adv Exp Med Biol. 457: 217–36. 10500797

[25] Schulze-Osthoff K, Ferrari D, Los M, Wesselborg S, Peter ME (1998) Apoptosis signaling by death receptors. Eur. J. Biochem. 254: 439–459. 10.1046/j.1432-1327.1998.2540439.x 9688254

[26] Micheau O, Tschopp J (2003) Induction of TNF receptor I-mediated apoptosis via two sequential signaling complexes. Cell 1142: 181–90. 10.1016/s0092-8674(03)00521-x 12887920

[27] Zou H, Li Y, Liu X, Wang X (1999) An APAF-1.cytochrome c multimeric complex is a functional apoptosome that activates procaspase-9. J Biol Chem. 274: 11549–56. 10.1074/jbc.274.17.11549 10206961

[28] Gupta S (2003) Molecular signaling in death receptor and mitochondrial pathways of apoptosis (Review). Int J Oncol. 22: 15–20. 12469180

[29] Zhu H, Huang M, Yang F, Chen Y, Miao ZH, et al (2007) R16, a novel amonafide analogue, induces apoptosis and G2-M arrest via poisoning topoisomerase II. Mol Cancer Ther. 6: 484–95. 10.1158/1535-7163.MCT-06-0584 17308047

[30] Cai Y, Lu J, Miao Z, Lin L, Ding J (2007) Reactive oxygen species contribute to cell killing and P-glycoprotein downregulation by salvicine in multidrug resistant K562/A02 cells. Cancer Biol Ther. 611: 1794–9. 10.4161/cbt.6.11.4860 18032928

[31] Rajendran P, Ho E, Williams DE, Dashwood RH (2011) Dietary phytochemicals, HDAC inhibition, and DNA damage/repair defects in cancer cells. Clin Epigenetics. 3: 4 doi:10.1186/1868-7083-3-4. 10.1186/1868-7083-3-4 PMC3255482 22247744

[32] Norbury CJ, Zhivotovsky B (2004) DNA damage-induced apoptosis. Oncogene 23: 2797–808. 15077143 10.1038/sj.onc.1207532

[33] Ljungman M (2010) The DNA damage response–repair or despair? Environ Mol Mutagen, 51: 879–89. Review. 10.1002/em.20597 20818630

[34] Tamm I, Wang Y, Sausville E, Scudiero DA, Vigna N, et al (1998) IAP-family protein survivin inhibits caspase activity and apoptosis induced by Fas (CD95), Bax, caspases, and anticancer drugs. Cancer Res 58: 5315–20. 9850056

[35] Shin S, Sung BJ, Cho YS, Kim HJ, Ha NC, et al (2001) An anti-apoptotic protein human survivin is a direct inhibitor of caspase-3 and -7. Biochemistry 40: 1117–23. 11170436 10.1021/bi001603q

[36] Chandele A, Prasad V, Jagtap JC, Shukla R, Shastry PR (2004) Upregulation of survivin in G2/M cells and inhibition of caspase 9 activity enhances resistance in staurosporine-induced apoptosis. Neoplasia 6: 29–40. 15068669 10.1016/s1476-5586(04)80051-4 PMC1679816

[37] Reed JC (2001) Apoptosis-regulating proteins as targets for drug discovery. Trends Mol Med 7: 314–9. 11425640 10.1016/s1471-4914(01)02026-3

[38] Vogel C, Hager C, Bastians H (2007) Mechanisms of Mitotic Cell Death Induced by Chemotherapy-Mediated G_2_ Checkpoint Abrogation. *Cancer Res* 67: 339–345. 17210716 10.1158/0008-5472.CAN-06-2548

[39] Vogelstein B, Lane D, Levine AJ (2000) Surfing the p53 network. Nature. 408: 307–10. 10.1038/35042675 11099028

[40] Selivanova G (2001) Mutant p53: the loaded gun. Curr Opin Investig Drugs, 2: 1136–41. Review. 11892926

[41] Fojo T (2002) p53 as a therapeutic target: unresolved issues on the road to cancer therapy targeting mutant p53. Drug Resist Updat. 55: 209–16. Review. 10.1016/s1368-7646(02)00119-x 12450786

[42] Bossi G, Lapi E, Strano S, Rinaldo C, Blandino G, et al (2006) Mutant p53 gain of function: reduction of tumor malignancy of human cancer cell lines through abrogation of mutant p53 expression. Oncogene 25: 304–9. 16170357 10.1038/sj.onc.1209026

[43] Wang X, Di Pasqua AJ, Govind S, McCracken E, Hong C, et al (2011) Selective depletion of mutant p53 by cancer chemopreventive isothiocyanates and their structure-activity relationships. J Med Chem. 54: 809–16. 10.1021/jm101199t PMC3139710 21241062

[44] Han J, Kim S, Yang JH, Nam SJ, Lee JE (2012) TPA-induced p21 expression augments G2/M arrest through a p53-independent mechanism in human breast cancer cells. Oncol Rep. 27: 517–22. 10.3892/or.2011.1511 22020547

[45] Sambucetti LC, Fischer DD, Zabludoff S, Kwon PO, Chamberlin H, et al (1999) Histone deacetylase inhibition selectively alters the activity and expression of cell cycle proteins leading to specific chromatin acetylation and antiproliferative effects. J Biol Chem. 274: 34940–7. 10.1074/jbc.274.49.34940 10574969

[46] Attoub S, Hassan AH, Vanhoecke B, Iratni R, Takahashi T, et al (2011) Inhibition of cell survival, invasion, tumor growth and histone deacetylase activity by the dietary flavonoid luteolinin human epithelioid cancer cells. Eur J Pharmacol. 651: 18–25. 10.1016/j.ejphar.2010.10.063 21074525

